# *Lrig1*-expressing epidermal progenitors require SCD1 to maintain the dermal papilla niche

**DOI:** 10.1038/s41598-023-30411-7

**Published:** 2023-03-10

**Authors:** Sophia Beng Hui Lim, Shang Wei, Andy Hee-Meng Tan, Maurice A. M. van Steensel, Xinhong Lim

**Affiliations:** 1 Institute of Medical Biology (IMB) / Skin Research Institute of Singapore (SRIS), Agency for Science, Technology and Research (A*STAR), 11 Mandalay Road, Clinical Sciences Building #17-01, Singapore, 308232 Republic of Singapore; 2 Bioprocessing Technology Institute (BTI), Agency for Science, Technology and Research (A*STAR), 20 Biopolis Way, Centros, Singapore, 138668 Republic of Singapore; 3 NUS Graduate School, National University of Singapore, Singapore, 119077 Republic of Singapore; 4 Lee Kong Chian School of Medicine, Nanyang Technological University, 11 Mandalay Road, Singapore, 308232 Republic of Singapore

**Keywords:** Cell biology, Stem cells

## Abstract

Niche cells are widely known to regulate stem/progenitor cells in many mammalian tissues. In the hair, dermal papilla niche cells are well accepted to regulate hair stem/progenitor cells. However, how niche cells themselves are maintained is largely unknown. We present evidence implicating hair matrix progenitors and the lipid modifying enzyme, Stearoyl CoA Desaturase 1, in the regulation of the dermal papilla niche during the anagen-catagen transition of the mouse hair cycle. Our data suggest that this takes place via autocrine Wnt signalling and paracrine Hedgehog signalling. To our knowledge, this is the first report demonstrating a potential role for matrix progenitor cells in maintaining the dermal papilla niche.

## Introduction

Skin niches regulate tissue-resident stem and progenitor cells by specifying cell fates^1,2^, replenishing niche cells^1,3^, and facilitating parent-daughter cell crosstalk in tissue regeneration^4,5^. A well-studied example is the dermal papilla (DP) of the hair follicle, a dynamic skin mini-organ that is regulated through reciprocal interactions between hair follicular epithelial cells and DP cells^6–15^. As an important stem cell niche, the DP plays pivotal roles in hair follicle maintenance and cycling^5,6,15–21^. It consists of mesenchymal cells that regulate the proliferation of hair follicle stem cells (HFSC) and germ (HG) progenitor cells, as well as promote the differentiation of their more mature progeny^19,22–24^. However, how DP cells themselves are sustained in vivo is not well understood.

Niches can influence stem and progenitor cell behaviour by initiating intercellular crosstalk by modulating the secretion of diffusible, inductive signals such as Wnt, Hedgehog and TGF-β ligands^14,20,25^. For instance, Wnt ligands secreted by epithelial matrix cells^11,19,26^ stimulate proliferation of HG progenitors^4,13,19^, by creating a Wnt permissive milieu via Rspondin induction in the DP^5^ during the transition from the hair resting (telogen) to growth (anagen)^19,27^. Sonic hedgehog (Shh) ligands expressed in matrix progenitor cells regulate DP maturation and maintenance^28–30^. TGF-β ligands, induced in the DP, induce apoptotic cell death in matrix progenitors to promote hair follicle regression (catagen)^20,31,32^. However, it is less clear if and how stem and progenitor cells may signal back to niche cells post-development.

Many of these signalling molecules, such as Wnt and Hedgehog, require palmitoylation for their activation and secretion^33^. This involves covalently linking ligands with palmitoleate, a monounsaturated fatty acid that is synthesized by the key lipid desaturating enzyme, SCD1^34,35^. Previous studies have demonstrated the importance of SCD1 for the maintenance of skin organs such as hair follicles and sebaceous glands (SG), since mice with germline^36^ or epidermal-specific^37^ knockout of *Scd1* lose their SG and hair. SCD1 is expressed most highly in SG cells, and the hair loss resulting from *Scd1* loss-of-function mutations has historically been attributed to the lack of sebum stemming from the absence of SGs. However, mice without differentiated sebocytes do not develop hair loss^38^, suggesting that differentiated sebocytes and sebum are not required for hair maintenance. This led us to hypothesize that the hair loss may instead be caused by the deletion of *Scd1* in the SG stem/progenitor cell compartment, which is contiguous with the junctional zone (JZ) and expresses *Lrig1*^39,40^.

Here, we tested this hypothesis by conditionally deleting *Scd1* in *Lrig1-Cre*^*ERT2/*+^ cells during the first telogen phase, and observed initially normal hair growth but subsequently hair loss. We obtained the *Lrig1-Cre*^*ERT2*^ mouse from The Jackson Labs, which is constructed differently from the *Lrig1-EGFP-Cre*^*ERT2*^ mouse reported by Page et al.^39^ and Jensen et al.^40^. This *Lrig1-Cre*^*ERT2*^ mouse has been reported to display greater efficiency of inducible Cre recombination activity compared to *Lgr-eGFP-IRES-Cre*^*ERT2*^ mice, possibly because the presence of the eGFP-IRES may make the translation of the Cre^ERT2^ less efficient. Accordingly, *Lrig1-Cre*^*ERT2*^ mice may similarly be more efficient at Cre recombination than *Lrig1-eGFP-IRES-Cre*^*ERT2*^ mice, and thus be more sensitive at marking and deleting in Lrig1^+ve^ cells. In addition to the JZ expression that was also reported by Page et al.^39^ and Jensen et al.^40^, our RNA in situ hybridization and lineage tracing data showed Lrig1^+ve^ cells and their labelled daughter cells in multiple epidermal compartments, including the hair follicle lower isthmus, telogen bulge, secondary hair germ and their anagen bulb matrix progeny. Consistent with previous studies, deletion of *Scd1* in *Lrig1-Cre*^*ERT2/*+^ cells resulted in ablation of the SG, though the hair follicle was still able to continue to grow and transition from telogen to anagen. However, the absence of *Scd1* in *Lrig1-Cre*^*ERT2/*+^ hair matrix cells eventually led to a cascade of events which culminated in progressive hair loss during the late anagen phase of adult mice. Our data suggest a model where *Scd1* is needed to regulate autocrine Wnt signalling in the matrix and paracrine Shh signalling to the DP cells. Degradation of the DP, in turn, may cause matrix progenitors to lose proliferative ability so that hair growth stops.

## Results

### ***Scd1*** is knocked out in ***Lrig1-Cre***^***ERT2/***+^ progeny in the interfollicular epidermis (IFE), junctional zone (JZ), isthmus (IS), sebaceous glands (SG) and hair bulge

**Figure 1:**
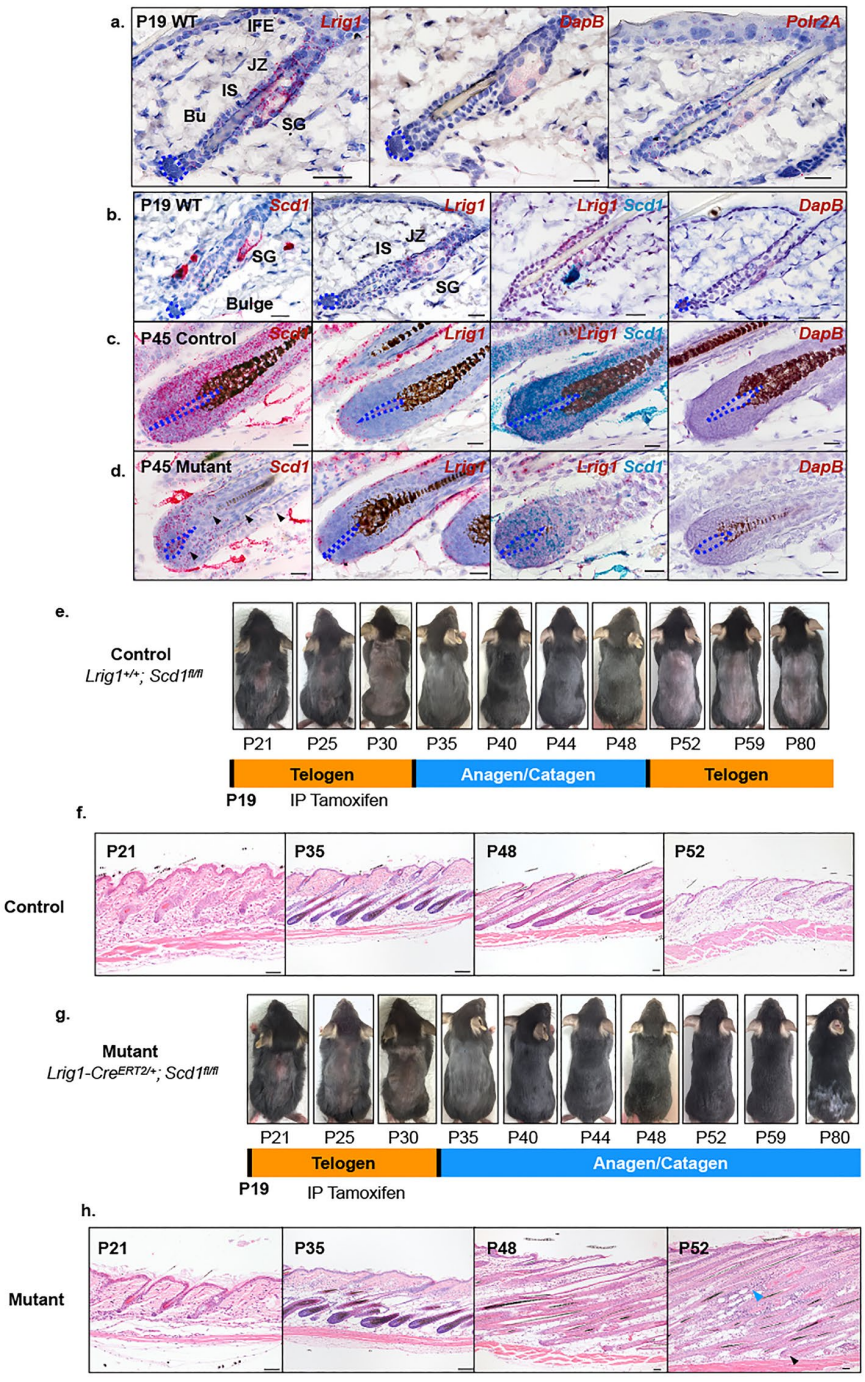
Phenotypic changes following *Scd1* deletion in *Lrig1*-expressing epidermal hair follicle cells. (**a**) RNA *in-situ* hybridisation of *Lrig1* on P19 WT dorsal skin. *Polr2A* (housekeeping gene) was used as positive control and *DapB* (Bacterial gene) as negative control. (**b**) RNA *in-situ* hybridisation of *Scd1*, *Lrig1* and Duplex *Lrig1* and *Scd1* on P19 WT Telogen hair follicle. (**c**) RNA *in-situ* hybridisation of *Scd1*, *Lrig1* and Duplex in P45 Control *Lrig1*^+*/*+^*; Scd1*^*fl/fl*^ anagen bulb. (**d**) RNA *in-situ* hybridisation of *Scd1*, *Lrig1* and Duplex in P45 Mutant *Lrig1-Cre*^*ERT2/*+^*; Scd1*^*fl/fl*^ anagen bulb. Purple dotted line denotes boundary of DP cells. Black arrowhead denotes loss of *Scd1* transcript expression (scale bar 20 μm). (**e**) Representative image of shaved Lrig1 Control mouse whole body hair cycle progression (n = 10 mice), post tamoxifen injection at P19. (**f**) H&E stained sections of Lrig1 Control skin at P21, P40, P48 and P52. (**g**) Representative image of shaved Lrig1 Mutant mouse whole body hair cycle progression (n = 13 mice), post tamoxifen injection at P19. (**h**) H&E stained sections of Lrig1 Mutant skin at P21, P40, P48 and P52. Blue arrowhead indicates presence of cellular infiltrates. Black arrowhead indicates exposed club-like hair shaft and missing DP (scale bar 50 μm).

We first verified the location *of Lrig1*-expressing cells in the skin using RNA in situ hybridisation at the first telogen phase. While we observed that *Lrig1* is enriched in the JZ, as originally described^40–42^, we also observed expression in the IFE, IS and the bulge, albeit at lower levels (Fig. 1a). Lineage tracing using *Lrig1-Cre*^*ERT2/*+^*; Rosa-mTmG* mice showed Lrig1^+ve^ progeny distributed throughout the epidermis, IS, hair bulge and outer lower bulge cells (secondary hair germ) at 2 days post treatment (Fig. S1a). While previous reports have suggested that Lrig1^+ve^ cells in the hair bulge are not CD34^+ve^ hair follicle stem cells^39,40^, careful examination of the data suggests that they are present in the outer bulge cells, something which others have not reported. Consistent with this, we detected Lrig1^+ve^ cells in the outer and lower bulge, including the secondary hair germ, when we administered a higher dose of unmetabolized tamoxifen just prior to the start of hair regeneration (Fig. S1a). After 1 month of tracing, these cells contributed not just to the SG but also to the IFE and all the different lineages of the hair follicle, including the matrix progenitors (Mx) (Fig. S1a). However, no Lrig1^+ve^ cells were observed in the DP. In addition, a few Lrig1^+ve^ cell progeny were detected in papillary dermal fibroblasts (Fig. S1a)^43,44^, and sporadically in intradermal adipocytes (Fig. S1b). Altogether, our data demonstrates that Lrig1^+ve^ progeny cells contribute to the IFE, JZ, IS, SG, hair bulge, matrix progenitors and differentiated cells of the mature hair follicle.

During telogen, *Scd1* mRNA is highly enriched in the SG and weakly expressed in the telogen hair bulge (Fig. 1b). During anagen, it is strongly expressed in the hair bulb (Fig. 1c). When *Scd1* deletion was induced in *Lrig1-Cre*^*ERT2/*+^ mice at P19, we observed that SGs completely disappeared by P25, 6 days post tamoxifen treatment (Fig. S2a,b). Nevertheless, the hair follicles continued to grow and transition from telogen to anagen (Fig. 1g,h), even though *Scd1* expression was greatly reduced in the P25 telogen hair bulge (Fig. S2a) and most of the P45 anagen hair bulb (Fig. 1d). Our data shows that deletion of *Scd1* in *Lrig1*-expressing cells leads to rapid loss of SGs, with no change in hair follicle morphology.

### Deletion of *Scd1* in *Lrig1-*expressing cells leads to abnormal “dysmorphic” hair follicles and progressive hair loss

Previous studies evaluating Scd1 deficiency reported abnormal hair cycling (prolonged anagen phase) and subsequent hair loss^36^. We observed that HFs progressed from telogen to anagen in *Lrig1-Cre*^*ERT2/*+^*; Scd1*^*fl/fl*^ mutant mice (Fig. 1g). To investigate the specific events leading up to the hair loss, we decided to examine the hair cycle more closely in mice that were shaved every two days. Despite reduced *Scd1* in the bulge (Fig. S2a), both control and mutant mice entered anagen from P35 onwards (Figs. 1e,g and S2e, injected mutant and control), suggesting that *Scd1* is not required for anagen entry in *Lrig1-*expressing cells. The timing of the hair cycles of both Lrig1 mutant and control differ from that described in the literature for male C57BL6 mice^45^, and may have resulted from the mixed 129S6/SvEvTac genetic background that we used (Fig. S2e, un-injected mutant and control), as well as the inhibitory effect that tamoxifen has on hair growth^46–48^ (Fig. S2e, injected mutant and control). Further tracking of the hair cycle revealed that, while control hair follicles progressed to telogen at P52 (Fig. 1e,f), mutant mice displayed a “dysmorphic” hair coat with fewer hair shafts at P80 (Figs. 1g and S2d). Histological examination of the mutant skin revealed the presence of narrow and elongated hair follicles lacking hair bulb structures that typically contain matrix progenitors and DP (Fig. S2c). Loss of both compartments eventually culminated in exposed “club-like” hair shafts that became surrounded by an intradermal inflammatory infiltrate (Figs. 1h and S2c). This abnormal hair phenotype seemed to present itself during the anagen-catagen transition phase (occurring from P48-52 in our mice), where the mouse coat appeared superficially to still be in anagen (Fig. S2e, injected mutant). The observation that SG loss in the mutant mice did not immediately cause abnormal hair cycling and growth suggests that the hair phenotype is independent of the loss of the SG and sebum, in contrast to what has been previously proposed^36^. These observations suggest that *Scd1* is required in *Lrig1*-expressing cells for hair follicle maintenance in late anagen, and hair cycle progression from anagen to catagen.

### Loss of *Scd1* in matrix progenitors leads to abrogation of DP marker expression

**Figure 2:**
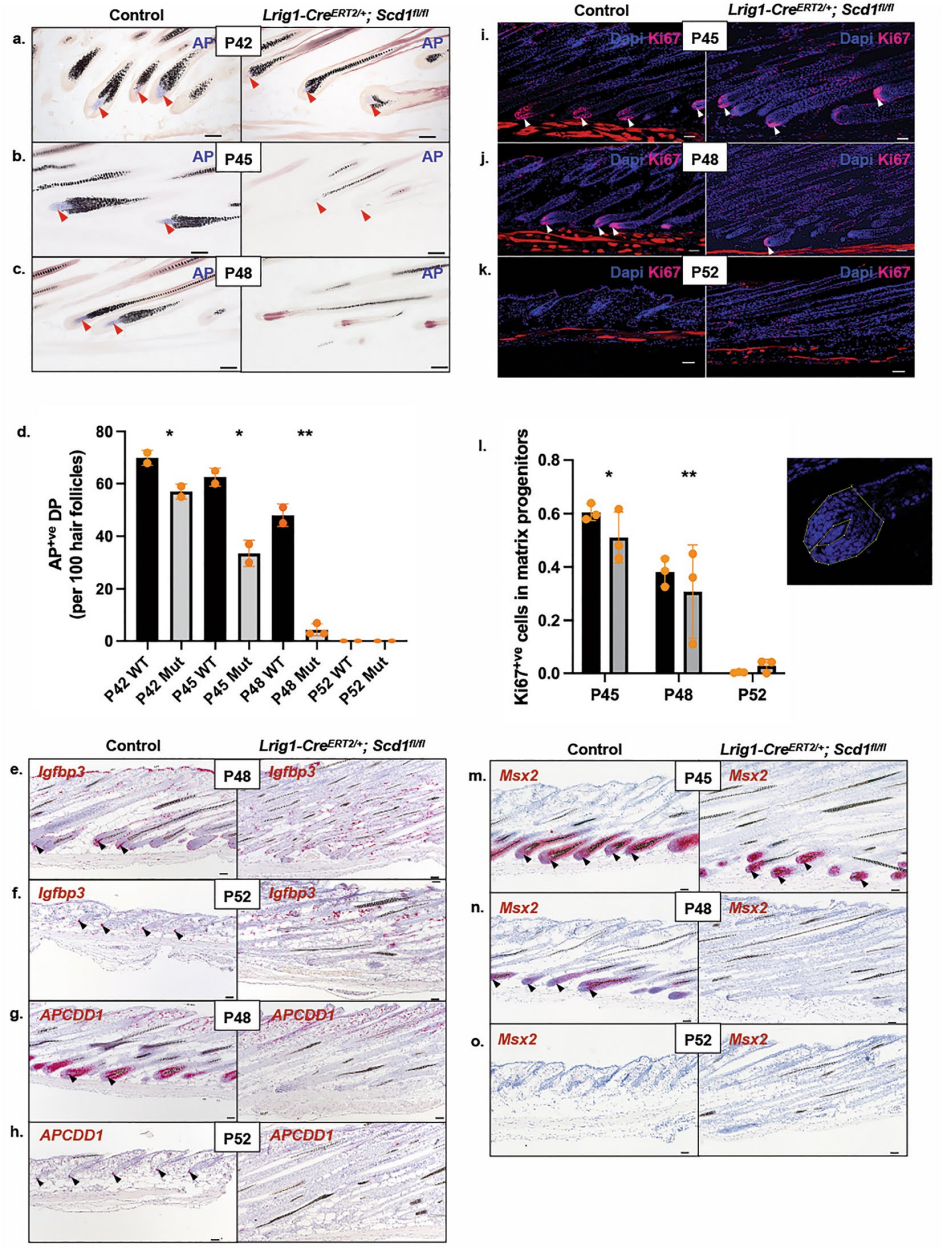
Altered expression of dermal papilla and hair matrix markers following *Scd1* deletion. (**a**–**c**) Representative images of alkaline phosphatase (AP) stain of Lrig1 Control and Mutant hair follicles at P42, P45 and P48. Red arrowhead indicates the presence of AP^+ve^ DP in anagen and catagen hair follicles. (**d**) Quantification of AP^+ve^ DP in Lrig1 Control and Mutant hair follicles at P42, P45, P48 and P52 (n = 2 mice, 100 hair follicles per mouse) Student's t-test applied, *p < 0.05 and **p < 0.001. (**e**,**f**) RNA *in-situ* hybridisation of *Igfbp3* in DP cells at P48 and P52. Black arrowhead indicates the presence of Igfbp3^+ve^ DP in hair follicles. (**g**,**h**) RNA *in-situ* hybridisation of *APCDD1* in DP cells at P48 and P52. Black arrowhead indicates the presence of APCDD1^+ve^ DP in hair follicles. (**i**–**k**) Ki67 antibody stained sections of Lrig1 control and Mutant skins at P45, P48 and P52. White arrowhead indicates Ki67^+ve^ matrix. (**l**) Percentage of Ki67^+ve^ cells in Lrig1 Control and Mutant matrix progenitors at P45, P48 and P52 (n = 3 mice, 12 hair follicles analysed per mouse). Student’s t-test applied, *p < 0.05 and **p < 0.001. Yellow opaque line denotes analysed area of expression. (**m**–**o**) RNA in-situ hybridisation of *Msx2* in matrix cells, IRS and pre-cortex of Lrig1 Control and Mutant skins at P45, P48 and P52. Black arrowhead indicates Msx2^+ve^ matrix cells (scale bar 50 μm).

Upon closer histological examination, we found that the hair follicles in mutant skin presented as abnormal, “dysmorphic” structures apparently lacking DPs (Figs. 1h and S2c). To test this notion, we stained the skins for Alkaline Phosphatase (AP), a widely accepted DP marker for anagen and catagen hair follicles^17,45,49^. We also probed for *Igfbp3* and *APCDD1*, which we had previously found to be highly expressed in DP cells throughout the hair cycle (unpublished data). Control hair DP exhibited well-defined AP staining from P42 to P48 during anagen and catagen (Fig. 2a–d). On the other hand, mutant hair DP stained positively for AP at P42 (Fig. 2a,d) but much more weakly at P45 (Fig. 2b,d) and not at all from P48 onwards (Fig. 2c,d). Consistent with this, *Igfbp3* and *APCDD1* were highly expressed in control but not mutant hair DP from P48 onwards (Figs. 2e–h and S3a–d). Despite the presence of αSMA^+ve^ DP precursors^50–53^ (Fig. S3u), mutant hair DP marker expression began to disappear at P45 (Fig. 2b,d). These data suggests that *Scd1* is required in *Lrig1*-expressing cells to maintain DP marker expression.

### Matrix cell proliferation is abrogated in the context of *Scd1* deletion-induced DP marker disappearance

Since the DP acts as a signalling centre that directs the surrounding matrix cells to proliferate^4,18,19,54^, we asked whether matrix cells were affected by *Scd1* deletion-induced DP degradation. Hair bulbs of control mice in early catagen at P48 showed strong Ki67 expression, while the majority of hair follicles in the mutant mice showed either reduced or no Ki67 staining (3.8-fold, *p* = 0.001) at the base (Fig. 2i,j,l). Neither control nor mutant hair follicles at P52 showed Ki67 staining (*p* = 0.0756) (Fig. 2k,l), though control follicles appeared to be in telogen while mutant follicles still appeared “dysmorphic”. In addition, we observed an absence of the matrix marker *Msx2*^55,56^ in mutant hair from P48 onwards (Figs. 2n,o and S3g,h). Similarly, at P52, the “dysmorphic” mutant hair still did not have any Msx2^+ve^ cells (Figs. 2o and S3i–j). TUNEL^+ve^ (*p* = 0.9701) and Cleaved Caspase-3^+ve^ (CC3) (*p* = 0.2302) cells were detected at the bottom of both control and mutant hair follicles from P48 onwards (Fig. S3p,m). Control hair follicles eventually lacked Msx2^+ve^, TUNEL^+ve^ (*p* = 0.4622) and CC3^+ve^ (*p* = 0.2341) cells because they were in telogen and did not have hair bulbs (Figs. 2o, S3p,m, respectively). Our data suggests that *Scd1* is required in *Lrig1*-expressing cells to regulate matrix progenitor cell proliferation and *Msx2* expression.

### *Scd1* deletion affects Wnt signalling in matrix progenitors

**Figure 3:**
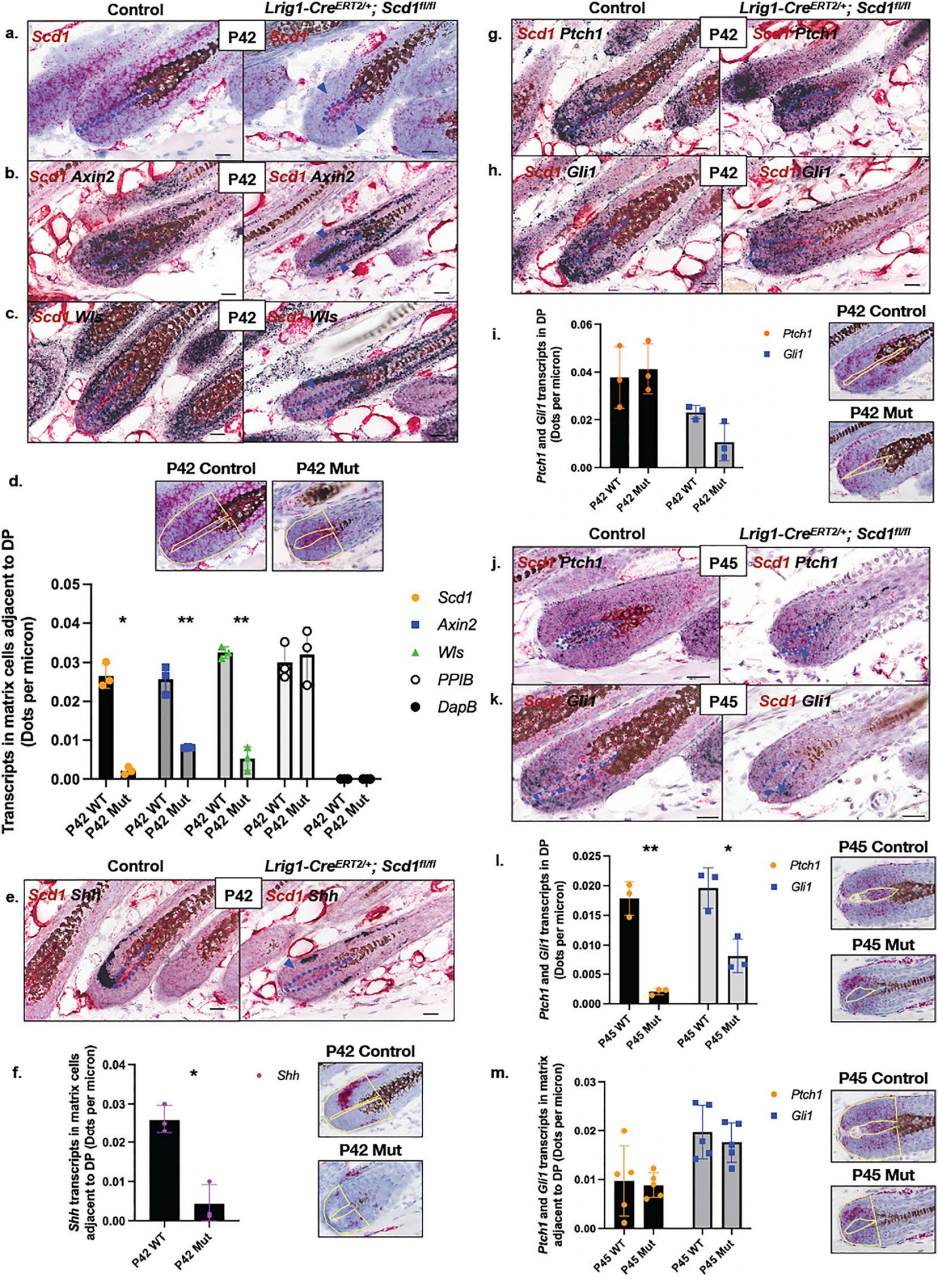
*Scd1*-deletion reduced expression of genes in key hair follicle signalling pathways, Wnt and Hedgehog. (**a**) RNA *in-situ* hybridisation of *Scd1* in Lrig1 Control and Mutant hair bulb at P42. Purple arrowhead indicates loss of *Scd1* transcript expression. (**b**) Duplex RNA *in-situ* hybridisation of *Scd1* and *Axin2* in Lrig1 Control and Mutant hair bulb at P42. Purple arrowhead indicates loss of *Axin2* transcript expression. (**c**) Duplex RNA *in-situ* hybridisation of *Scd1* and *Wls* in Lrig1 Control and Mutant hair bulb at P42. Purple arrowhead indicates loss of *Wls* transcript expression. (**d**) *Scd1*, *Axin2*, *Wls*, *PPIB* and *DapB* transcripts by spot detection in P42 Lrig1 Control and Mutant mice (n = 3 animals, 12 hair follicles analysed per animal). *p < 0.05 and **p < 0.001. Yellow solid line indicates area of representative image analysed for transcripts. (**e**) Duplex RNA *in-situ* hybridisation of *Scd1* and *Shh* in Lrig1 Control and Mutant hair bulb at P42. Purple arrowhead indicates loss of *Shh* transcript expression. (**f**) *Shh* transcripts by spot detection in adjacent matrix of P42 Lrig1 Control and Mutant mice (n = 3 animals, 12 hair follicles analysed per animal). *p < 0.05 and **p < 0.001. Yellow solid line indicates area of representative image analysed for transcripts. (**g**) Duplex RNA *in-situ* hybridisation of *Scd1* and *Ptch1* in Lrig1 Control and Mutant hair bulb at P42. (**h**) Duplex RNA *in-situ* hybridisation of *Scd1* and *Gli1* in Lrig1 Control and Mutant hair bulb at P42. Purple dotted line denotes boundery of DP cells. Purple arrowhead indicates loss of *Shh/Ptch1/Gli1* expression and area analysed (scale bar 20 μm). (**i**) *Ptch1* and *Gli1* transcripts by spot detection in DP of P42 Lrig1 Control and Mutant mice (n = 3 animals, 12 hair follicles analysed per animal). *p < 0.05 and **p < 0.001. Yellow solid line indicates area of representative image analysed for transcripts. (**j**) Duplex RNA *in-situ* hybridisation of *Scd1* and *Ptch1* in Lrig1 Control and Mutant hair bulb at P45. Purple arrowhead indicates loss of *Ptch1* transcript expression. (**k**) Duplex RNA *in-situ* hybridisation of *Scd1* and *Gli1* in Lrig1 Control and Mutant hair bulb at P45. Purple arrowhead indicates loss of *Gli1* transcript expression. (**l**) *Ptch1* and *Gli1* transcripts by spot detection in DP of P45 Lrig1 Control and Mutant mice (n = 3 animals, 12 hair follicles analysed per animal). *p < 0.05 and **p < 0.001. Yellow solid line indicates area of representative image analysed for transcripts. (**m**) *Ptch1* and *Gli1* transcripts by spot detection in Matrix of P45 Lrig1 Control and Mutant mice (n = 3 animals, 12 hair follicles analysed per animal). *p < 0.05 and **p < 0.001. Yellow solid line indicates area of representative image analysed for transcripts. Purple dotted line denotes boundary of DP cells (scale bar 20 μm).

Given that Wnt signalling is involved in hair stem cell maintenance^57–60^ and that Wnt ligands require palmitoylation for Wnt activation^33^, we hypothesized that *Scd1* ablation might affect *Msx2* expression in matrix cells by altering Wnt signalling in matrix progenitors. We found that Wnt signalling is significantly downregulated prior to DP degradation at P45. Using RNA in situ hybridisation, we observed that the expression of *Axin2*, a well-known Wnt target gene^57,61^, was significantly reduced (2.85-fold, *p* = 0.0002) in P42 matrix progenitors where *Scd1* was ablated (threefold, *p* = 0.0138), while it remained highly expressed in areas where *Scd1* was still present (Figs. 3a,b,d and S5a,b). Consistent with reduced *Axin2* expression, the expression of *Wls*, another Wnt target gene^13^, was also significantly reduced (1.54-fold, *p* = 0.0004) in matrix cells after *Scd1* deletion (Fig. 3c,d). Our data suggests that *Scd1* deletion in *Lrig1*-expressing cells reduces Wnt signalling in matrix progenitors.

Matrix cells express a variety of Wnts^58,62^, suggesting that matrix cell Wnt signalling may proceed in an autocrine manner. To determine if reduced Wnt activity in matrix progenitors could be attributed to the deficiency of specific ligands or receptors, we surveyed the expression of 17 Wnt ligands (Fig. S4a–q) and 10 Frizzled receptors (Fig. S4r–z) using RNA in situ hybridisation. We found that the expression of Wnt ligands, particularly in the matrix (Fig. S4f,g,i,k,n,o), and receptors was largely similar in control and mutant skin (Fig. S4r–z). Taken together, our data suggests that reduced Wnt signalling in matrix progenitors is not due to changes in Wnt ligand and receptor expression.

### *Scd1* ablation is followed by the loss of *Shh* in matrix progenitors and paracrine Shh signalling in the DP

Several studies have shown that Wnt activation can induce Hedgehog signalling in hair follicles^63–65^ where Shh signalling is known to regulate hair morphogenesis and hair cycling^11,29,66–68^, and HFSC-associated events^28,30^. Post-translational palmitoylation also seems to enhance secretion of Hedgehog ligands^69–73^. Therefore, we next examined whether Hedgehog signalling was affected by *Scd1* deletion in the matrix. Following *Scd1*-mediated Wnt attenuation, *Shh* expression was significantly reduced (fourfold, *p* = 0.0035) in mutant hair matrix cells neighbouring the DP at P42 (Fig. 3e,f), while there was no significant change in the expression of Hedgehog target genes *Ptch1* (*p* = 0.7255) and *Gli1* (*p* = 0.0594) in mutant hair matrix and DP cells (Fig. 3g,h,i). This was followed at P45 by a significant loss of *Ptch1* (sixfold, *p* = 0.0007) and *Gli1* (1.6-fold, *p* = 0.0113) despite the presence of *Scd1* in the DP (Fig. 3j,k,l), whereas *Gli1* and *Ptch1* remained expressed in *Scd1-*deleted matrix cells (Fig. 3j,k,m). *Ihh* and *Dhh* were not expressed in either control or mutant skins at P45 (Fig. S6a,b), including SGs (Fig. S6e–g). These data suggests that *Scd1* deletion in *Lrig1*-expressing cells leads to the loss of Shh expression in matrix progenitors, and Hh signalling in the DP.

## Discussion

Although it is well established that the DP, as an important skin niche, promotes the maintenance of stem and progenitor cells in the hair follicle^2,4,27,59^, what sustains the DP in turn remains largely obscure. Our findings collectively support a model where matrix progenitors derived from Lrig1^+ve^ JZ and outer lower bulge cells (secondary hair germ) (Fig. S7a,b) require *Scd1* to regulate DP cells and enable anagen-catagen transition (Fig. S7c–e).

Consistent with the aberrant hair cycle phenotype reported in mice with germline^36^ and epidermal-specific (*K14Cre*) deletion of *Scd1*, we found that *Lrig1Cre*^*ERT2/*+^*; Scd1*^*fl/fl*^ mice exhibit abnormal “dysmorphic” hair with exposed hair shafts that gradually stopped growing during the second hair cycle. This phenotype suggests that *Scd1* deletion in *Lrig1-*expressing cells leads to abnormal “dysmorphic” hair and progressive hair loss. In addition, we found that the expression of DP markers in *Lrig1Cre*^*ERT2/*+^*; Scd1*^*fl/fl*^ hair follicles disappeared at P45 (Fig. 2b) prior to the loss of proliferative Msx2^+ve^ matrix cells at P48 and stalling of hair growth in the late anagen phase (Fig. 2i,m). These data show that *Scd1-*deletion affects DP and matrix marker expression, as well as matrix cell proliferation in mutant hair follicles. As we did not detect retention of hair fibres in the upper hair follicles (Fig. S8), we conclude that exposure of club-like hair shafts at the follicle base was not the result of accumulated inner root sheath (IRS) hair fibres and sebum loss, as previously proposed^36^. Moreover, immune cell infiltrates appeared much later, after DP marker expression was lost and when hair shafts became exposed (Fig. S3s). This observation suggests that inflammation is not the reason why the DP markers are lost. Considering that immunosuppressive factors are thought to be expressed in hair matrix cells^60,61^, we propose that intradermal inflammation likely occurred after DP marker disappearance and hair bulb shrinkage, and thus could not have caused the bulb to diminish.

We have interpreted the mutant hair follicles as “dysmorphic”, and surmise that the phenotype occurs during the anagen to catagen transition because the molecular and signalling pathway changes occur in our animals at P45, when the hair coat and follicles clearly still exhibit anagen morphology and characteristics such as proliferative matrix. However, while the majority of Lrig1 mutant hair follicles remained in an abnormal “dysmorphic” phase, we detected some follicles with catagen-like morphology at P48 (Fig. 2i) that appear to still have some DP-like structures attached. While we believe that this could be the result of incomplete Cre-mediated deletion of *Scd1* in *Lrig1*-expressing cells, we cannot exclude the possibility that some abnormal mutant hairs may have actually managed to transition into early catagen and then remained trapped in that state. Regardless, the follicles appear unable to transition any further, and we detect no DP-like structures in P52 mutant skin.

The DP is generally thought to communicate with matrix cells to promote hair growth and specify cell lineages in the hair follicle^2,4,13,59^. We found that the expression of Wnt ligands, particularly in the matrix (Fig. S4f,g,i,k,n,o), and receptors was largely similar in control and mutant skin (Fig. S4r–z). Our data suggest that loss of *Scd1* activity, rather than changes in Wnt ligand/receptor expression, disrupts autocrine Wnt/β-catenin signalling in matrix progenitors. Consistently, we found that Shh ligand expression in matrix progenitors was abolished following decreased Wnt signalling at P42 (Fig. 3e,f), just prior to DP cell degradation at P45. This was subsequently followed by attenuation of Hedgehog signalling in the DP, when it began to degenerate at P45 (Fig. 3j–l). We speculate that *Lrig1*-expressing matrix progenitors rely on Scd1 to regulate autocrine Wnt signalling and paracrine Hedgehog signalling.

Our lineage tracing data show Lrig1^+ve^ matrix progenitors derived from Lrig1^+ve^ ancestor cells in the JZ, bulge and outer lower bulge (secondary hair germ) (Fig. S1a). Our data also indicate that Lrig1^+ve^ cells can be found in the (A) IFE, (B) dermal fibroblast and (C) intradermal adipocyte compartments (Fig. S1a,b). (A) Most *Lrig1-*expressing IFE and (B) dermal fibroblasts reside in the upper papillary dermis (Fig. S1a), distant from the mature DP. We did not detect Hh ligand expression in the IFE and papillary dermis, where Lrig1^+ve^ fibroblasts reside (Fig. S6e,f). Moreover, the DP appears unaffected in late anagen hair follicles of *Pdgfra-CreER; Smo*^*fl/fl*^ mice^74^. (C) While intradermal adipocytes have been shown to stimulate hair growth through secreted adipogenic factors^75–77^, genetic ablation of mature and adipocyte precursors did not affect DP cells during development and post-natal maintenance^77^. Furthermore, *K14*-mediated *Scd1* knockout mice lose hair despite having intact *Scd1* in the skin adipocyte compartment^37^. Taken together, these observations suggest that Lrig1^+ve^ IFE keratinocytes, papillary dermal fibroblasts and intradermal adipocytes are unlikely to mediate Hedgehog signalling in our mutant mice. While Hedgehog signals can have both short- and long-range activity, we speculate that the matrix cells located adjacent to the DP are the most likely source of Hedgehog ligands here. It would be ideal to functionally test this using genetic mouse models for matrix-specific hedgehog ligand deletion but that is beyond the scope and resources of the present study.

While it is well recognised that the niche signals to and regulates stem and progenitor cells, the signals that maintain the niche and the sources of those signals remain poorly understood. Our data indicate that epithelial progenitor cells could signal back to their niches by providing at least one source of ligands to activate the Hedgehog signalling that DP cells may rely on for its maintenance and survival (Fig. 3j–l). The apparent requirement for this signal during the anagen-catagen transition raises several questions: how do progenitor cells sense hair cycle transitions and produce the right ligands? Does the niche only require the pro-survival signal at one point in the hair cycle, and if so, why? If not, what are the other signals and cells that promote niche survival? Are these signals conserved in other niche systems? Answers to these questions would yield new insights into the relationship between stem cells and their niches.

## Materials and methods

### Experimental animals

*Lrig1-Cre*^*ERT2/*+^^74^, *Scd1*^*fl/fl*^^76^ and *Rosa26-mTmG*^77^ mice have been described previously. *Lrig1-Cre*^*ERT2/*+^ and *Rosa26-mTmG* mice were obtained from The Jackson Laboratory, and *Scd1*^*fl/fl*^ mice were a gift from Dr James M. Ntambi, University of Wisconsin-Madison. All knockout and lineage tracing experiments were performed in postnatal day 19 (P19) animals. For knockout experiments in *Lrig1-Cre*^*ERT2/*+^ mice, a single dose of tamoxifen dissolved in corn oil (4 mg/ml/25 g body weight) was administered via intraperitoneal injection. Bioethics Council along with A*STAR Animal Care and Use Committee (IACUC no. 130883) approved the experimental study. We confirm that this study was performed in accordance with the guidelines and regulation of Bioethics Council and A*STAR Animal Care and Use Committee, and reported in accordance with ARRIVE guidelines.

### Histology and immunostaining

Animals were sacrificed using CO_2_ asphyxiation followed by cervical dislocation. Skins were removed from the dorsal region. For paraffin sections, tissues were fixed in 4% PFA overnight at room temperature with shaking. Tissues were then washed in phosphate buffered saline (PBS) and dehydrated through a series of ethanol baths, followed by immersion in xylene and paraffin wax. Tissues were cut into 7-μm thick sections using a Leica RM2255 microtome (Leica Microsystems). Sections were rehydrated and counterstained with Hematoxylin and Eosin where specified.

For frozen sections, tissues were fixed in 4% PFA overnight on the roller at 4 °C, washed in PBS, and stored overnight in 30% sucrose (w/v) at 4 °C. Tissues were embedded in OCT medium and stored at − 80 °C, and then sectioned at varying thicknesses using a Leica CM3050S cryostat (Leica Microsystems).

For immunostaining, frozen 7–10 μm thick sections were washed in PBS and incubated in blocking buffer (2% normal goat serum (catalogue no. 005-000-121, Jackson ImmunoResearch) and 0.2% Triton X in PBS) for 1 h at room temperature, then incubated with primary antibody diluted in blocking buffer overnight at 4 °C. This was followed by incubation with secondary antibody diluted in blocking buffer for 1 h at room temperature and mounting in Prolong Gold with DAPI mounting medium (catalogue no. P-36931, Life Technologies). All washes were performed using PBS. The following antibodies were used: Rat monoclonal anti-Ki67 (catalogue no. 14-5698-82, eBioscience), Rabbit polyclonal anti-mouse Scd1(M38) (catalogue no. #2438, Cell signalling), and Goat anti-Rabbit conjugated to Alexa Fluor A568 (catalogue no. A11036, Life Technologies). The following dyes were used: LipidTOX Green neutral lipid stain (catalogue no. H34475, Life Technologies), Phalloidin Alexa Fluor 568 (catalogue no. A12380, Life Technologies).

### Alkaline phosphatase stain

Fixed frozen tissues were sectioned at 10-16 μm thickness using a Leica CM3050S cryostat (Leica Microsystems). Frozen sections were washed in PBS and primed with Tris–EDTA pH9.1 for 5 min at room temperature, followed by incubation in AP solution (5 ml Tris–EDTA pH9.1, 33 µl of NBT and 16.5 µl of BCIP from Promega) for 5–7 min in room temperature. Tissues were then washed in deionized water and dehydrated in solutions of increasing ethanol concentration (70%, 80%, 90%, and 3 times at 100%), followed by xylene prior to mounting with Cytoseal (Thermo Scientific).

### RNA in situ hybridisation

Skin was harvested and fixed in 4% PFA for 20–24 h at room temperature, dehydrated and then embedded in paraffin. Tissue sections were cut at 7-μm thickness, air-dried at room temperature, and processed for RNA in situ detection using the RNAscope 2.5HD Red Detection assay and RNAscope2.5HD Duplex assay according to the manufacturer’s instructions (Advanced Cell Diagnostics). RNAscope probes used were as follows: *Scd1*, *Axin2*, *Wls*, *Wnt1*, *Wnt2*, *Wnt2b*, *Wnt3*, *Wnt3a*, *Wnt4*, *Wnt5a*, *Wnt5b Wnt6*, *Wnt7a Wnt7b*, *Wnt9a*, *Wnt9b*, *Wnt10a*, *Wnt10b*, *Wnt11*, *Wnt16*, *Fzd1*, *Fzd3*, *Fzd4*, *Fzd5*, *Fzd6*, *Fzd7*, *Fzd8*, *Fzd9*, *Fzd10*, *Shh*, *Gli1*, *Ptch1*, all of which were detected using Fast Red and HRP-based black detection reagent.

### Microscope imaging

All sections were imaged using Olympus FV3000 RS Inverted Confocal and Zeiss AxioImager microscopes. Image processing was performed with Fiji software version 1.0 (written by Wayne Rasband).

### Statistical analysis of immunohistochemistry staining

For RNA transcript quantification, transcript/dots within the region of interest (DP or hair matrix) were measured by area factor analysis. Dots per micron were calculated by dividing the area of red/black color spots by the area of each region of interest. For quantification of Ki67 +ve (magenta) cells within the hair bulb, the percentage of Ki67 +ve cells were calculated by area factor analysis, by dividing the area of magenta spots/cells (Ki67 + ve) by number of nuclei within the bulb matrix.

To evaluate statistical significance, all measurements were pooled for each animal, with mean and SEM values calculated. Statistical analyses were generated using Prism (Graphpad, Prism 9 version 9.3.1) to perform unpaired Student t tests, with statistically significant scores determined by p-values below 0.05 (*P < 0.05) and very statistically significant scores by p-values below 0.001 (**P < 0.001).

## Supplementary Information

Supplementary Information.

## Data Availability

The datasets generated and/or analysed during the current study are available in the MGI repository, MGI: 7286459, 7287840 and 3773308).
