# Author Correction: *Lrig1*-expressing epidermal progenitors require SCD1 to maintain the dermal papilla niche

**DOI:** 10.1038/s41598-023-32932-7

**Published:** 2023-04-07

**Authors:** Sophia Beng Hui Lim, Shang Wei, Andy Hee-Meng Tan, Maurice A. M. van Steensel, Xinhong Lim

**Affiliations:** 1 Institute of Medical Biology (IMB)/Skin Research Institute of Singapore (SRIS), Agency for Science, Technology and Research (A*STAR), 11 Mandalay Road, Clinical Sciences Building #17‑01, Singapore, 308232 Republic of Singapore; 2 Agency for Science, Technology and Research (A*STAR), Bioprocessing Technology Institute (BTI), 20 Biopolis Way, Centros, Singapore, 138668 Republic of Singapore; 3 NUS Graduate School, National University of Singapore, Singapore, 119077 Republic of Singapore; 4 Lee Kong Chian School of Medicine, Nanyang Technological University, 11 Mandalay Road, Singapore, 308232 Republic of Singapore

Correction to: *Scientific Reports* 10.1038/s41598-023-30411-7, published online 10 March 2023

The Acknowledgements section in the original version of this Article was incomplete. It now reads:

“We thank the following colleagues for their support and valuable suggestions: Dr Ang Siang Yun, Dr Keith Tan, Dr Kwa Mei Qi, Alvin Tan, and Dr Ivo De Vos. We thank the AMPL/Histology facility for performing Masson’s trichrome staining. This work was supported by Biomedical Research Council (BMRC), Agency for Science, Technology, and Research in Singapore (A*STAR); BMRC-A*STAR-EDB IAF-PP for the Skin Research Institute of Singapore (H17/01/a0/004), Acne and Sebaceous Gland Program (H17/ H17/01/a0/008), and National Medical Research Council Clinician-Scientist Individual Research Grant MOH-CIRG20nov-0009.”

The original Article has been corrected.

