# Supplementary material for: Lrig1-expressing epidermal progenitors require SCD1 to maintain the dermal papilla niche
Source: Sci Rep. 2023 Mar 10;13:4027. doi: 10.1038/s41598-023-30411-7 (PMC10006094; doi:10.1038/s41598-023-30411-7)
Supplement: Supplementary file 1 — Supplementary Information. [file 41598_2023_30411_MOESM1_ESM.pdf]

a.

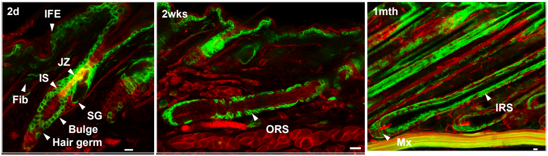

2d
IFE
JZ
IS
Fib
SG
Bulge
Hair germ
2wks
ORS
1mth
IRS
Mx

b.

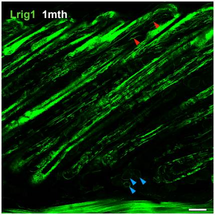

Lrig1 1mth

Figure S1: Lrig1^{+ve} cells contribute to epidermis, isthmus, hair bulge, hair germ, matrix progenitors, some papillary dermal fibroblasts and few intradermal adipocytes during anagen

a) Lineage tracing of Lrig1^{+ve} progenies from *Lrig1-Cre$^{ERT2/+}$; Rosa-mTmG* mice over 2 days, 2 weeks and 1 month from P19. White arrowheads denote Lrig1^{+ve} progenies.

b) Lineage tracing of *Lrig1^{+ve}* progenies from *Lrig1-Cre$^{ERT2/+}$; Rosa-mTmG* mice at 1 month from P19 induction. Red arrowheads denote *Lrig1^{+ve}* papillary dermal fibroblasts. Blue arrowheads indicate *Lrig1^{+ve}* intradermal adipocytes. (Scale bar: 20μm)

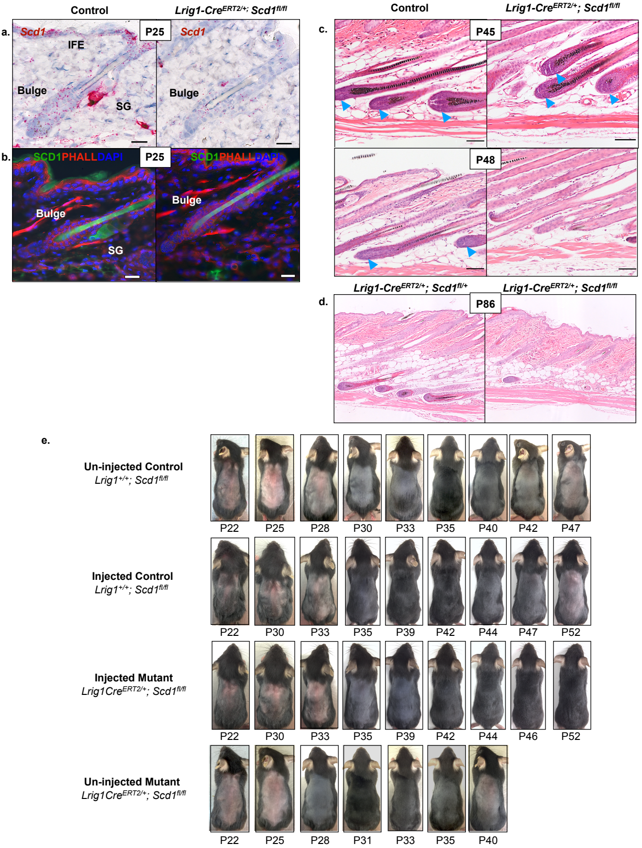

Control
Lrig1-CreERT2/+; Scd1fl/fl
a.
Scd1
IFE
P25
Scd1
Bulge
SG
Bulge
b.
SCD1PHALLDAPI
P25
SCD1PHALLDAPI
Bulge
SG
Bulge
Control
Lrig1-CreERT2/+; Scd1fl/fl
c.
P45
P48
Lrig1-CreERT2/+; Scd1fl/+
Lrig1-CreERT2/+; Scd1fl/fl
d.
P86
e.
Un-injected Control
Lrig1+/+; Scd1fl/fl
P22 P25 P28 P30 P33 P35 P40 P42 P47
Injected Control
Lrig1+/+; Scd1fl/fl
P22 P30 P33 P35 P39 P42 P44 P47 P52
Injected Mutant
Lrig1CreERT2/+; Scd1fl/fl
P22 P30 P33 P35 P39 P42 P44 P46 P52
Un-injected Mutant
Lrig1CreERT2/+; Scd1fl/fl
P22 P25 P28 P31 P33 P35 P40

Figure S2: Phenotypic changes following Scd1 deletion in *Lrig1*-expressing epidermal hair follicle cells

a) RNA *in-situ* hybridisation of *Scd1* in Lrig1 Control and Mutant telogen hair follicle at P25.

b) SCD1 and Phalloidin (PHALL) Antibody stained sections of Lrig1 Control and Mutant skins at P25 (Scale bar: 20μm)

c) H&E stain skin sections of Lrig1 Control and Mutant mice at P45 and P48 at 20X magnification. Light blue arrowhead indicates the presence of hair bulb (Scale bar: 50μm)

d) H&E stain skin sections of Lrig1 Het and Mutant mice at P86 (Scale bar: 50μm)

e) Hair cycle of Tamoxifen injected and un-injected Lrig1 Control and Mutant mice.

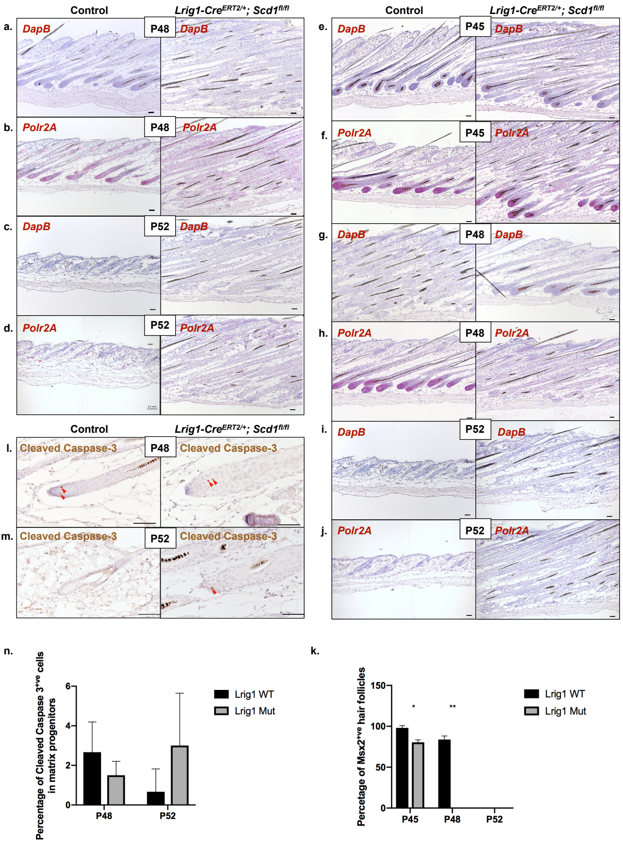

Control
Lrig1-CreERT2/+; Scd1fl/fl
a.
DapB
P48
DapB
b.
Polr2A
P48
Polr2A
c.
DapB
P52
DapB
d.
Polr2A
P52
Polr2A
e.
DapB
P45
DapB
f.
Polr2A
P45
Polr2A
g.
DapB
P48
DapB
h.
Polr2A
P48
Polr2A
i.
DapB
P52
DapB
j.
Polr2A
P52
Polr2A
l.
Cleaved Caspase-3
P48
Cleaved Caspase-3
m.
Cleaved Caspase-3
P52
Cleaved Caspase-3
n.
Percentage of Cleaved Caspase 3+ve cells in matrix progenitors
6
4
2
0
P48
P52
Lrig1 WT
Lrig1 Mut
k.
Percetage of Msx2+ve hair follicles
150
100
50
0
*
**
P45
P48
P52
Lrig1 WT
Lrig1 Mut

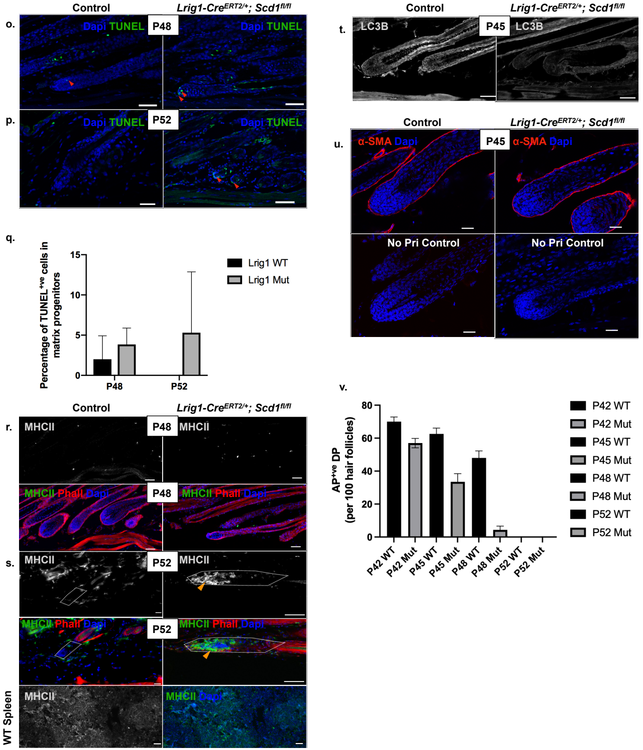

Control
Lrig1-Cre^ERT2/+; Scd1^fl/fl
o.
Dapi TUNEL
P48
Dapi TUNEL
p.
Dapi TUNEL
P52
Dapi TUNEL
q.
Percentage of TUNEL +ve cells in matrix progenitors
15
10
5
0
P48
P52
Lrig1 WT
Lrig1 Mut
Control
Lrig1-Cre^ERT2/+; Scd1^fl/fl
r.
MHCII
P48
MHCII
MHCII Phall Dapi
P48
MHCII Phall Dapi
s.
MHCII
P52
MHCII
MHCII Phall Dapi
P52
MHCII Phall Dapi
WT Spleen
MHCII
MHCII Dapi
Control
Lrig1-Cre^ERT2/+; Scd1^fl/fl
t.
LC3B
P45
LC3B
Control
Lrig1-Cre^ERT2/+; Scd1^fl/fl
u.
α-SMA Dapi
P45
α-SMA Dapi
No Pri Control
No Pri Control
v.
AP+ve DP (per 100 hair follicles)
80
60
40
20
0
P42 WT
P42 Mut
P45 WT
P45 Mut
P48 WT
P48 Mut
P52 WT
P52 Mut

Figure S3: Altered expression of dermal papilla and hair matrix markers following Scd1 deletion

(a-d) RNA *in-situ* hybridisation of positive and negative controls of *Igfbp3* and *APCDD1* in Lrig1 Control and Mutant at P48 and P52.

(e-j) RNA *in-situ* hybridisation of positive and negative controls of *Msx2* in Lrig1 Control and Mutant at P45, 48 and P52 (Scale bar: 50μm)

(k) Percentage of $Msx2^{+ve}$ hair follicles in Lrig1 Control and Mutant hair at P45, P48 and P52 (n= 3 animals, 12 hair follicles analysed per animal).

(l and m) Cleaved Caspase-3 (CC3) expressions in Lrig1 Control and Mutant hair bulbs at P48 and P52. Red arrowheads denote apoptotic cells.(Scale bar: 50μm)

(n) Cleaved Caspase 3 expression by area factor analysis in Lrig1 Control and Mutant matrix progenitors at P48 and P52 (n=3 mice). *$p < 0.05$ and **$p < 0.001$.

(o and p) TUNEL expressions in Lrig1 Control and Mutant hair bulbs at P48 and P52. Red arrowheads denote apoptotic cells.

(q) Percentage of $TUNEL^{+ve}$ cells in Lrig1 Control and Mutant matrix progenitors (n=3 animals, 12 hair follicles analysed per animal).*$p < 0.05$ and **$p < 0.001$.

(r and s) MHCII and PHALL antibody staining of Lrig1 Control and Mutant skin section at P48 and P52. Orange arrowheads denote $MHCII^{+ve}$ cells around hair follicles. White dotted line marks hair bulb of mutant follicles and lower follicle of the control mice in telogen. Positive control of MHCII in WT mouse spleen.

(t) LC3B antibody staining for autophagy in Lrig1 Control and Mutant skin section at P45.

(u) α-SMA stained section of Lrig1 Control and Mutant hair follicles at P45. (Scale bar: 50μm)

(v) Quantification of AP^{+ve} DP in Lrig1 Control and Mutant hair follicles at P42, P45, P48 and P52 (n=2 mice, 100 hair follicles per animal)

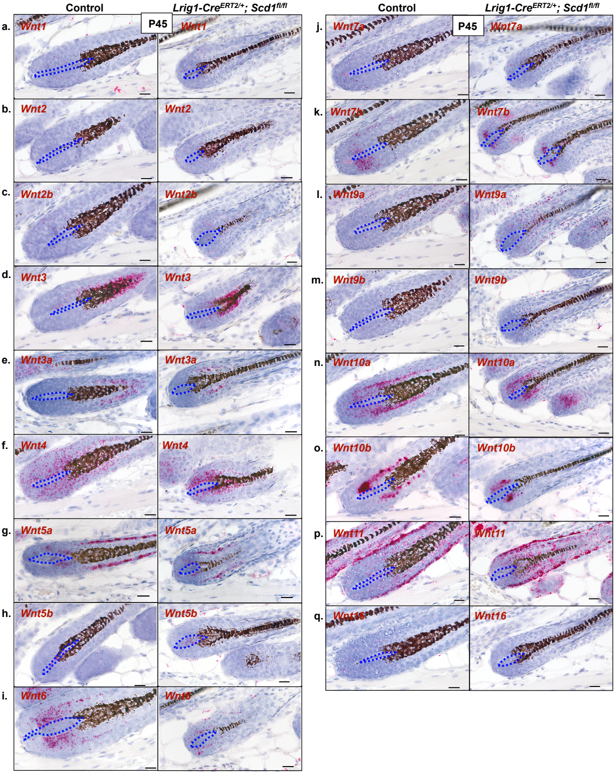
Control
Lrig1-CreERT2/+; Scd1fl/fl
P45
a. Wnt1
b. Wnt2
c. Wnt2b
d. Wnt3
e. Wnt3a
f. Wnt4
g. Wnt5a
h. Wnt5b
i. Wnt6
j. Wnt7a
k. Wnt7b
l. Wnt9a
m. Wnt9b
n. Wnt10a
o. Wnt10b
p. Wnt11
q. Wnt16

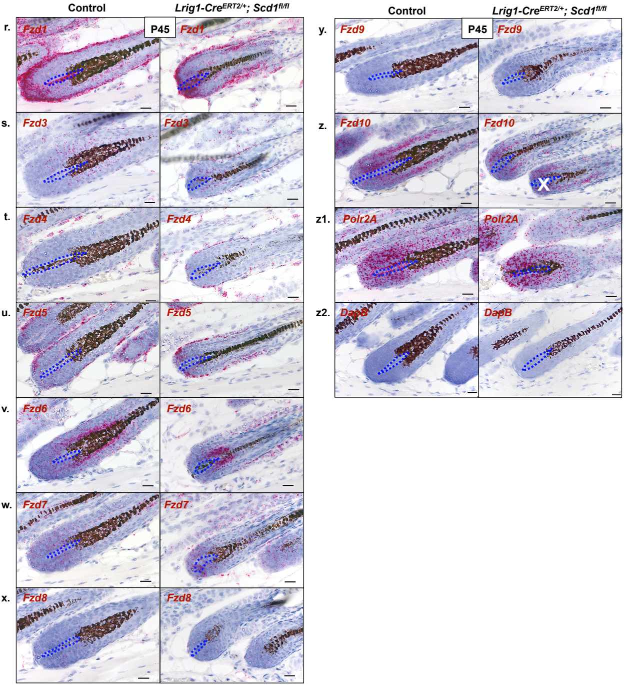
Control
Lrig1-CreERT2/+; Scd1fl/fl
P45
r.
Fzd1
Fzd1
s.
Fzd3
Fzd3
t.
Fzd4
Fzd4
u.
Fzd5
Fzd5
v.
Fzd6
Fzd6
w.
Fzd7
Fzd7
x.
Fzd8
Fzd8
Control
Lrig1-CreERT2/+; Scd1fl/fl
P45
y.
Fzd9
Fzd9
z.
Fzd10
Fzd10
X
z1.
Polr2A
Polr2A
z2.
DapB
DapB

Figure S4: Loss of Wnt signalling in epithelial matrix progenitors not due to expression changes of Wnt ligand and receptors

(a-q) RNA *in-situ* hybridisation of Wnts in hair bulbs at P45.

(r-z) RNA *in-situ* hybridisation of Frizzled receptor in hair bulbs at P45.

(z1 and z2) RNA *in-situ* hybridisation of positive (*Polr2A*) and negative control (*DapB*) at P45. Purple dotted lines denote approximate boundary of DP cells. (Scale bar: 20μm)

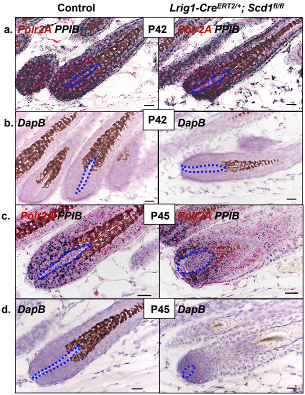
Control
Lrig1-Cre$^{ERT2/+}$; Scd1$^{fl/fl}$
a.
Polr2A PPIB
P42
Polr2A PPIB
b.
DapB
P42
DapB
c.
Polr2A PPIB
P45
Polr2A PPIB
d.
DapB
P45
DapB

Figure S5: RNA *in-situ* hybridisation control expressions

(a) RNA *in-situ* hybridisation of positive controls (*Polr2A* and *PPIB*) at P42.
(b) RNA *in-situ* hybridisation of negative control (*DapB*) at P42.
(c) RNA *in-situ* hybridisation of positive control (*Polr2A* and *PPIB*) at P45.
(d) RNA *in-situ* hybridisation of negative control (*DapB*) at P45.

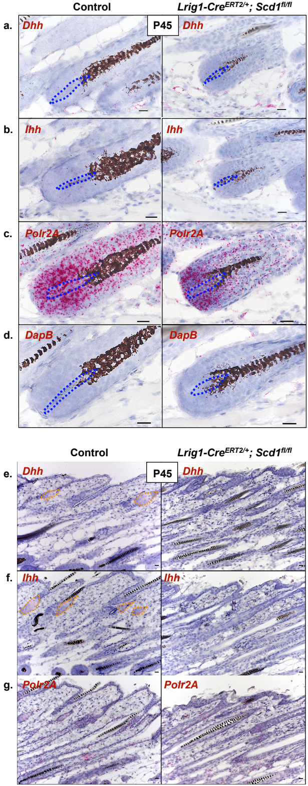
Control
Lrig1-Cre$^{ERT2/+}$; Scd1$^{fl/fl}$
P45
a.
Dhh
b.
Ihh
c.
Polr2A
d.
DapB
Control
Lrig1-Cre$^{ERT2/+}$; Scd1$^{fl/fl}$
P45
e.
Dhh
f.
Ihh
g.
Polr2A

Figure S6: Deletion of *Scd1* in *Lrig1*-expressing cells did not change *Ihh* or *Dhh* expression

(a) RNA *in-situ* hybridisation of *Dhh* in Lrig1 Control and Mutant hair bulbs at P45.
(b) RNA *in-situ* hybridisation of *Ihh* in Lrig1 Control and Mutant hair bulbs at P45.
(c and d) RNA *in-situ* hybridisation of positive (*Polr2A*) and negative control (*DapB*) at P45. Purple dotted lines denote approximate boundary of DP cells.
(e) RNA *in-situ* hybridisation of *Dhh* in Lrig1 Control and Mutant sebaceous glands at P45.
(f) RNA *in-situ* hybridisation of *Ihh* in Lrig1 Control and Mutant sebaceous glands at P45.
(g) RNA *in-situ* hybridisation of positive (*Polr2A*) in Lrig1 Control and Mutant sebaceous glands at P45. Orange dotted lines mark sebaceous glands (Scale bar: 20μm)

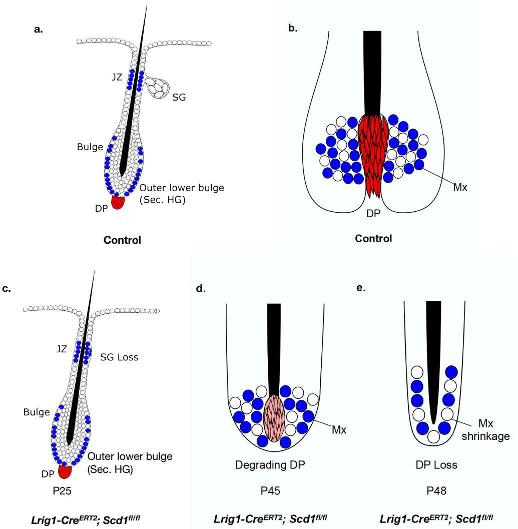
a.
JZ
SG
Bulge
Outer lower bulge
(Sec. HG)
DP
Control
b.
Mx
DP
Control
c.
JZ
SG Loss
Bulge
Outer lower bulge
(Sec. HG)
DP
P25
Lrig1-CreERT2; Scd1fl/fl
d.
Mx
Degrading DP
P45
Lrig1-CreERT2; Scd1fl/fl
e.
Mx
shrinkage
DP Loss
P48
Lrig1-CreERT2; Scd1fl/fl

Figure S7: Proposed model of Scd1-mediated matrix signalling and DP survival

(a) Lrig1^{+ve} cells (blue circles) in Control telogen hair follicle at P19.

(b) Model: Lineage tracing of Lrig1^{+ve} junctinal zone cells (blue circles) to matrix progenitors.

(c-e) Schematic diagram of phenotype progression in Lrig1 mutants: SG loss at P25, DP degradation at P45 and matrix shrinkage at P48.

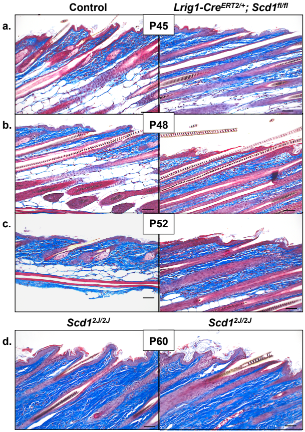

Control
Lrig1-CreERT2/+; Scd1fl/fl
a.
P45
b.
P48
c.
P52
Scd12J/2J
Scd12J/2J
d.
P60

Figure S8: No cornified plugs observed in pilary canals of Lrig1 Mutant hair follicles

(a-c) Masson trichome staining of Lrig1 Control and Mutant skin sections at P45, P48 and P52.

(d) Masson trichome staining of *Asebia*$^{2J/2J}$ dorsal skin at P60. (Scale bar: 20μm)

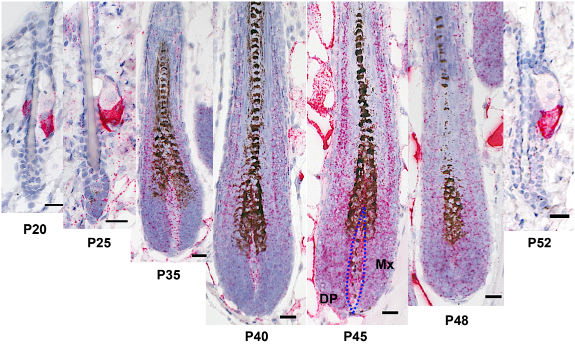
P20
P25
P35
P40
Mx
DP
P45
P48
P52

Figure S9: ***Scd1*** **is increasingly expressed in the hair bulb and peaks during late anagen**

RNA *in-situ* hybridisation of *Scd1* in WT dorsal hair follicles during hair cycling. (Scale bar: 20μm)
